# Aromatase Inhibitors as Adjuvant Therapy in Early Breast Cancer: Insights into Toxicities and Their Management

**DOI:** 10.3390/cancers17172726

**Published:** 2025-08-22

**Authors:** Simone Nardin, Beatrice Ruffilli, Tommaso Lupo Landolfo, Giulia Isingrini, Ida Taglialatela, Andrea Delbarba, Francesca D’Avanzo, Valentina Rossi, Eduardo Celentano, Benedetta Conte, Matteo Nardin, Alessandra Gennari

**Affiliations:** 1 Division of Medical Oncology, Maggiore University Hospital, 28100 Novara, Italy; 2 Department of Internal Medicine and Medical Sciences (DiMI), School of Medicine, University of Genova, 16126 Genova, Italy; 3 Department of Translational Medicine, University of Piemonte Orientale, 28100 Novara, Italy; 4 Department of Clinical and Experimental Sciences, SSD of Endocrinology, ASST Spedali Civili of Brescia, 25123 Brescia, Italy; 5 Department of Electrophysiology, Humanitas Gavazzeni, Via Mauro Gavazzeni 21, 24125 Bergamo, Italy; 6 Department of Biomedical Sciences, Humanitas University, Via Rita Levi Montalcini 4, 20090 Pieve Emanuele-Milan, Italy; 7 Internal Medicine, Department of Medicine, ASST Spedali Civili di Brescia, Piazzale Spedali Civili 1, 25123 Brescia, Italy

**Keywords:** aromatase inhibitors, breast cancer survivorship, endocrine therapy toxicity, cardio-oncology, supportive cancer care

## Abstract

Aromatase inhibitors (AIs), with or without gonadotropin-releasing hormone analogs, are the cornerstone of adjuvant endocrine therapy for women with hormone receptor-positive early-stage breast cancer, offering significant reductions in recurrence risk and improving long-term survival. Their use is frequently accompanied by treatment-related toxicities that can adversely affect patients’ quality of life (QoL) and adherence to therapy. Commonly reported side effects include vasomotor symptoms, such as hot flashes; musculoskeletal disorders, such as arthralgia and myalgia; mood disorders; and genitourinary discomfort, such as vaginal dryness and dyspareunia. Additionally, AIs are associated with a heightened risk of bone loss, leading to osteoporosis and fractures, and may have implications for cardiovascular health. Effective management of these adverse events is pivotal in maintaining treatment adherence and preserving QoL. Evidence-based strategies to address these toxicities include pharmacological interventions, such as analgesics for joint pain, bisphosphonates or denosumab for bone health, and hormonal or non-hormonal approaches for vasomotor and genitourinary symptoms. Non-pharmacological measures, including physical activity, dietary adjustments, and complementary therapies, can also help mitigate symptoms. This review examines the broad spectrum of AI-associated toxicities, discusses their clinical implications, and provides an overview of evidence-based management strategies. These insights aim to support clinicians in optimizing patient care while minimizing the toxicities of therapy.

## Simple Summary

Aromatase inhibitors are a cornerstone of adjuvant treatment for women with hormone-sensitive breast cancer, helping to reduce the risk of the disease returning and improving survival. However, long-term use is often accompanied by side effects such as joint pain, hot flashes, fatigue, bone loss, sexual problems, and cardiovascular issues. These complications can greatly affect quality of life and may lead some patients to stop treatment early. This review summarizes the most common side effects, explains their causes, and outlines both medical and lifestyle strategies to prevent or manage them. Integrating the latest scientific evidence with practical recommendations may support a more personalized and proactive approach to care, helping patients maintain both treatment effectiveness and overall well-being. Future directions in research are also highlighted to further improve tolerability and quality of life in breast cancer survivors.

## 1. Introduction

Breast cancer (BC) is the most commonly diagnosed cancer worldwide, accounting for 11.7% of all new cancer cases [1]. Among its various subtypes, hormone receptor-positive (HR+) BC, defined by estrogen receptor and/or progesterone receptor expression ≥ 1%, is the most prevalent [2,3]. Endocrine therapy with tamoxifen or aromatase inhibitors (AIs) remains the cornerstone of adjuvant treatment for early-stage HR+ BC, with a standard treatment duration of five years [4,5,6]. In recent years, accumulating evidence has supported extending endocrine therapy beyond five years, particularly in patients with a higher initial risk of relapse [6,7].

In clinical practice, determining the optimal type and duration of endocrine therapy is a nuanced, multifactorial process that must consider several factors, including menopausal status, recurrence risk, potential side effects, and the overall toxicity profile of the chosen treatment [8]. Importantly, AIs are associated with a range of adverse effects, such as vasomotor disturbances, musculoskeletal symptoms, and genitourinary complaints, which can significantly impair the patient’s quality of life (QoL). These toxicities are among the most common reasons for non-adherence and early discontinuation of therapy [9,10,11,12,13]. 

This review aims to comprehensively explore the spectrum of toxicities associated with AI use in the adjuvant setting for HR+ early BC and to present current evidence-based strategies for their management. A comprehensive literature search was performed using the PubMed/MEDLINE, Scopus, and ClinicalTrials.gov databases, covering articles published up to June 2025. The following keywords and combinations were used: “aromatase inhibitors”, “endocrine therapy”, “early breast cancer”, “toxicity”, “adverse effects”, “side effects”, “management”, “fatigue”, “bone loss”, “vasomotor symptoms”, “sexual dysfunction”, “musculoskeletal symptoms”, and “cognitive impairment”. Articles were selected based on relevance to clinical practice and level of evidence, with particular attention to randomized controlled trials, meta-analyses, guideline recommendations, and recent expert reviews. By addressing both pharmacological and non-pharmacological interventions, this study seeks to support clinicians in minimizing treatment-related burdens and enhancing adherence and long-term outcomes.

## 2. Mechanism of Action

**Figure 1:**
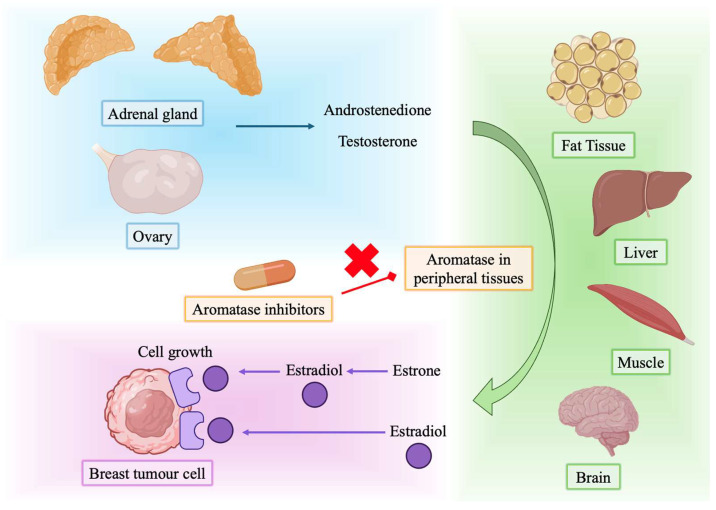
Aromatase pathway and its inhibition by aromatase inhibitors in peripheral tissues.

AIs exert their primary therapeutic effect by targeting and inhibiting aromatase (CYP19A1), a cytochrome P450 monooxygenase that plays a crucial role in estrogen biosynthesis [14,15]. This enzyme catalyzes the aromatization of C19 androgens through three sequential hydroxylation steps and demethylation of the androgen A-ring, converting androstenedione and testosterone to estrone and estradiol, respectively [16,17]. In postmenopausal women, peripheral tissues such as adipose tissue, muscle, skin, bone, and even breast tumors become the primary sites of estrogen production, and inhibition of aromatase in these tissues leads to a systemic reduction in circulating estrogen (Figure 1) [18]. AIs are classified into two main categories: steroidal and non-steroidal. Steroidal inhibitors, such as exemestane, are structural analogs of androstenedione that bind irreversibly to the substrate-binding site of the aromatase enzyme, resulting in permanent inactivation.

In contrast, nonsteroidal inhibitors, such as anastrozole and letrozole, bind reversibly to the heme group of the enzyme [19]. The irreversible mechanism of steroidal AIs confers the benefit of sustained suppression even after serum drug levels decline, whereas reversible inhibitors require continuous presence for their effect [20]. This potent estrogen suppression underlies the clinical use of HR+ BC. By significantly reducing both systemic and intratumoral estrogen levels, AIs prevent estrogen receptor activation in neoplastic cells, thereby inhibiting proliferation and reducing the risk of recurrence [21]. Several landmark trials have demonstrated the superiority of AIs over tamoxifen in postmenopausal women, showing approximately 30% lower recurrence rates and improved survival [4,5].

However, estrogen plays a ubiquitous role beyond breast tissue, serving as a regulator of the metabolic, skeletal, vascular, neurological, and reproductive systems. Thus, the profound reduction in estrogen levels (to approximately 10–15% of pre-AI levels) disrupts hormonal homeostasis and contributes to the diverse toxicity profile associated with AI therapy [22].

Vasomotor symptoms (VMS) are strongly related to hormonal homeostasis. Estrogen plays a pivotal role in thermoregulation via the hypothalamus, and its decline is believed to narrow the thermoneutral zone, contributing to symptoms such as hot flashes and night sweats, frequently reported with AI use [23,24].

Musculoskeletal symptoms, particularly arthralgia and myalgia, are prevalent among AI-treated patients and appear to be of multifactorial origin. Estrogen modulates nociceptive pathways, collagen synthesis, and inflammatory cytokine activity; its deficiency is associated with the upregulation of pro-inflammatory mediators and heightened pain sensitivity, potentially leading to the joint pain and stiffness observed in 30–50% of users [25,26,27]. Moreover, genetic polymorphisms affecting estrogen signaling or metabolism may contribute to individual susceptibility to these symptoms [28].

Bone health is one of the most well-established domains affected by estrogen deprivation. Estrogen is essential for maintaining the balance between osteoclast-mediated bone resorption and osteoblast-driven bone formation. It promotes osteoblast survival and function while inhibiting osteoclastogenesis by modulating the receptor activator of nuclear factor kappa B/receptor activator of nuclear ligand pathway. Estrogen depletion shifts bone remodeling toward net resorption and results in measurable reductions in bone mineral density over time [29,30]. Clinical trials have consistently demonstrated higher rates of osteoporosis and fractures in AI-treated women than in those treated with tamoxifen, which has partial estrogen agonist effects on the bone [5,31].

Genitourinary and sexual dysfunction, such as vaginal dryness, dyspareunia, and decreased libido, are also strongly associated with the hypoestrogenic state induced by AIs. Estrogen is essential for maintaining the trophic state of the vaginal epithelium and promoting lubrication and elasticity. Its absence results in epithelial thinning, loss of lubrication, and increased susceptibility to irritation and discomfort, substantially affecting sexual health and QoL [32,33,34].

Cognitive and mood-related changes have been reported anecdotally and in some prospective studies, although the data remain heterogeneous. Estrogen exerts neuroprotective effects by influencing neurotransmitter systems, such as serotonin and acetylcholine, and supporting hippocampal neurogenesis. Therefore, estrogen withdrawal may impact cognition and mood, although confounding variables, including age, menopausal status, and cancer-related psychological stress, complicate attribution [35,36].

Fatigue, one of the most reported and difficult-to-quantify adverse effects, likely reflects an interplay between endocrine disruption, chronic inflammation, and indirect effects from associated symptoms such as insomnia, pain, or depression. Although mechanistic clarity is lacking, estrogen deficiency may contribute to altered cytokine expression and mitochondrial dysfunction, thereby contributing to reduced energy levels and increased fatigue in AI-treated patients [37,38].

The cardiovascular (CV) effects of AIs are more complex and somewhat controversial. Estrogen has cardioprotective effects through its favorable effects on lipid profiles, endothelial function, and vascular tone. AI therapy can lead to increased LDL cholesterol, decreased HDL cholesterol, and potential endothelial dysfunction, theoretically increasing CV risk, particularly in patients with pre-existing risk factors [39,40]. However, head-to-head comparisons with tamoxifen suggest a nuanced picture, as tamoxifen’s favorable lipid effects may magnify the apparent CV disadvantage of AIs [5,41].

## 3. General Management of Side Effects

AIs are associated with a broad range of adverse effects that may significantly impair QoL and lead to treatment discontinuation. Effective management of these toxicities is essential to ensure long-term therapeutic success. This section provides a comprehensive overview of the most common side effects encountered during AI therapy and outlines both pharmacological and non-pharmacological strategies for their mitigation. A summary of the principal toxicities is presented in Table 1, including categorization of each intervention by recommendation level (first-line, second-line, investigational) and a qualitative assessment of evidence strength (high, moderate, low).

### 3.1. Vasomotor Symptoms

VMS, particularly hot flashes, are among the most frequently reported adverse effects of endocrine therapy, affecting 30–95% of women undergoing treatment for BC. Their incidence is strongly influenced by menopausal status [104]. The pathophysiology of VMS is primarily attributed to estrogen deficiency, which disrupts the hypothalamic thermoregulation pathways [105].

A patient-centered approach is essential for managing AI-induced VMS, aiming to balance symptom relief and oncologic safety. For mild-to-moderate symptoms, non-pharmacological strategies are recommended as first-line interventions [53,54,106,107,108]. These include lifestyle modifications such as dressing in layers, avoiding known triggers such as spicy foods, caffeine, and alcohol, and maintaining regular physical activity [109]. Although generally safe, these measures have shown only modest efficacy. Weight management appears to play an important role in reducing VMS severity [55]. Several studies, including those by Caan et al., suggest that intentional weight loss of ≥10% is associated with a clinically meaningful reduction in hot flash frequency [54,110]. Additionally, data from the WHEL study support dietary interventions, particularly low-fat, high-fiber diets, as potentially beneficial for reducing VMS prevalence [111]. Beyond symptom control, weight management contributes to improved cancer outcomes, CV health, and overall QoL [112,113].

Psychological interventions, such as cognitive behavioral therapy (CBT), mindfulness-based techniques, paced respiration, and clinical hypnosis, have also been shown to be effective in reducing VMS-related distress [53,106,108,114]. CBT has consistently been shown to reduce the perceived burden and daily interference of VMS, even when the symptom frequency remains unchanged. In the MENOS 1 trial, CBT significantly reduced VMS-related bother compared to usual care, with improvements sustained for 26 weeks [42]. Further evidence supporting CBT in this setting comes from a large multicenter randomized trial by Duijts et al., which evaluated both CBT and physical exercise in patients experiencing treatment-related menopausal symptoms. At 12 weeks, patients in the CBT group reported significantly lower levels of perceived burden from hot flashes and night sweats than those receiving usual care, with effects maintained at the 6-month follow-up [43]. Additionally, CBT led to sustained improvements in daily functioning, sleep quality, and overall QoL in women experiencing menopausal symptoms following BC treatment [42].

Clinical hypnosis has emerged as a promising non-pharmacological option for managing VMS in BC survivors. Randomized controlled trials suggest that hypnosis can significantly reduce both the frequency and severity of hot flashes while improving QoL. In a pivotal study, Elkins et al. reported up to a 68% reduction in hot flash symptom scores following hypnosis, along with notable improvements in patient-reported interference with daily functioning [58]. Additional support comes from a randomized pilot study by MacLaughlan et al., which compared standardized hypnotherapy with gabapentin in a cohort of 27 BC survivors. Participants receiving hypnotherapy experienced an 80% median reduction in hot flash frequency and an 85% reduction in symptom severity after eight weeks of treatment, with minimal side effects and high adherence [59].

Emerging procedural interventions, such as the stellate ganglion block (SGB), have shown initial promise for refractory VMS. Both randomized controlled and uncontrolled trials have reported that SGB can result in substantial reductions in hot flash frequency and intensity compared with pregabalin or other techniques, with effect sizes ranging from 50 to 70% and benefits sustained for up to six months [60,61]. Additionally, improvements in sleep quality and overall tolerability were noted, with a favorable safety profile.

In contrast, although mind–body interventions, such as yoga, have been investigated for the alleviation of VMS, current evidence regarding their efficacy remains mixed. Although consistent reductions in hot flash frequency have not been universally demonstrated, yoga has been associated with improvements in related domains such as sleep quality, mood, and psychological well-being, supporting its role as a supportive care modality [115].

Acupuncture has also been investigated as a complementary approach for managing VMS in BC survivors, particularly in those pursuing non-pharmacological options. Several randomized controlled trials and meta-analyses suggest that acupuncture may significantly reduce the frequency and severity of hot flashes. However, the findings are heterogeneous, with some studies demonstrating superiority over sham procedures and others reporting limited benefits [44]. A meta-analysis by Zhang et al. indicated that acupuncture was associated with a significant reduction in hot flashes compared to sham procedures [45]; however, in a previous meta-analysis, acupuncture did not appear to provide a significant benefit in decreasing the frequency or intensity of hot flashes [46]. Although more rigorous trials are needed to define optimal treatment protocols and duration, acupuncture is generally well tolerated and may be considered a safe adjunctive option for women who prefer integrative therapies or have contraindications to pharmacological interventions [47,48].

Despite the benefits of non-pharmacological interventions, a subset of patients with BC continues to experience moderate-to-severe VMS that significantly impairs their QoL. In these cases, pharmacological therapies may be warranted to achieve adequate symptom control, provided their use is guided by careful consideration of oncological safety and individual patient risk profiles.

Management of these symptoms is particularly challenging, as hormone replacement therapy (HRT) is generally contraindicated in this patient population [116,117]. Consequently, non-hormonal agents such as selective serotonin reuptake inhibitors (SSRIs), serotonin-norepinephrine reuptake inhibitors (SNRIs), gabapentinoids, and centrally acting agents like clonidine have become the mainstays of treatment. Among antidepressants, SSRIs and SNRIs have shown powerful activity in reducing the frequency and severity of hot flashes. Venlafaxine is the most studied agent in this setting, showing a 61% reduction in VMS among patients with BC, with a rapid onset of action typically observed within the first weeks of treatment. The therapeutic effect of venlafaxine appears to be dose-dependent; indeed, 37.5 mg/day can yield modest symptom relief (40% reduction) compared to higher doses (75 mg/day or 150 mg/day) [118]. Loprinzi et al. suggested that venlafaxine should start with a daily dose of 37.5 mg/day and could be increased to 75 mg/day, but no higher, given the increased rate of side effects [49]. In addition, in women who experienced a 50% decrease in objectively measured hot flashes, treatment with venlafaxine was associated with beneficial effects on secondary endpoints, such as reduced fatigue, enhanced sleep quality, increased vitality, and improved psychological well-being [50].

Clonidine, a 2-adrenergic agonist, was one of the earliest non-hormonal options investigated for VMS. A randomized controlled trial conducted by the University of Rochester Cancer Center demonstrated that oral clonidine (0.1 mg/day) reduced the frequency of hot flashes by 37% at 4 weeks compared to 20% in the placebo group [119]. However, when compared to venlafaxine in a crossover study, clonidine and venlafaxine achieved similar median reductions in hot flash scores of 55% and 49%, respectively. In contrast, clonidine had fewer discontinuations due to adverse events [51].

The anticonvulsant gabapentin has shown comparable efficacy, with a 46% reduction in hot flashes at a 900 mg/day dose [52]. A randomized crossover trial demonstrated comparable efficacy between gabapentin and venlafaxine, with both treatments achieving approximately a 66% reduction in hot flash frequency compared to placebo. However, when patient treatment preferences were evaluated, venlafaxine was favored over gabapentin, suggesting greater overall acceptability [120].

Oxybutynin has recently been evaluated as a non-hormonal treatment for hot flashes. The ACCRU SC-1603 randomized trial showed improvement in hot flashes and QoL compared to the placebo group; however, long-term safety, particularly regarding the cognitive effects of anticholinergic use, remains unexamined and warrants further investigation [121].

A new class of non-hormonal agents, neurokinin receptor antagonists, is reshaping the landscape of VMS treatment by targeting hypothalamic kisspeptin–neurokinin B–dynorphin (KNDy) neurons. Among these, fezolinetant and elinzanetant have shown promising efficacy and tolerability in recent studies. Fezolinetant, a selective neurokinin-3 receptor (NK3R) antagonist, has demonstrated robust efficacy in reducing the frequency and severity of moderate-to-severe VMS in phase III trials. Specifically, in the SKYLIGHT 1 study, daily treatment with fezolinetant 30 mg or 45 mg produced rapid symptom relief detectable within the first week, with over 50% reduction in VMS by week 12, sustained through 52 weeks of follow-up [56]. Improvements in menopause-specific QoL measures were also observed, particularly in the domains related to physical and psychosocial functioning. However, it is important to note that SKYLIGHT 1 excluded patients with a history of BC, limiting the generalizability of its findings to the oncology population. To address this gap, the ongoing HIGHLIGHT 1 trial (NCT06440967) is specifically evaluating the safety and efficacy of fezolinetant in women with ER + BC experiencing VMS due to endocrine therapy.

Elinzanetant, a dual NK1R/NK3R antagonist, appears to offer even greater symptom relief, as reported by the recently published OASIS-4 trial evaluating the efficacy and safety of this molecule in a specific population of patients with ER+ BC undergoing endocrine therapy [57]. Cardoso et al. demonstrated a statistically significant reduction in the daily frequency of moderate-to-severe VMS compared to placebo at 12 weeks, alongside notable improvements in sleep disturbances and menopause-related QoL. These data collectively position both fezolinetant and elinzanetant as attractive nonhormonal alternatives for BC survivors experiencing AI-induced VMS, especially given their rapid onset and avoidance of estrogen-related safety concerns.

The role of systemic HRT in VMS relief among women with a history of BC remains highly contested. The Stockholm randomized controlled trial, with over 10 years of follow-up, found no overall increase in recurrence or mortality among BC survivors receiving combined estrogen–progestogen therapy; however, a statistically significant increase in contralateral BC was observed [122]. Conversely, a systematic review and meta-analysis by Poggio et al. reported that HRT use was associated with a nearly 46% increased risk of BC recurrence, particularly in those with ER+ tumors [123]. Consequently, current guidelines generally contraindicate systemic HRT in BC survivors [109,124]. Nonetheless, for patients with refractory symptoms and low-risk tumor profiles, HRT may be considered after an individualized risk-benefit assessment and shared decision-making.

### 3.2. Musculoskeletal Symptoms

Aromatase-induced musculoskeletal symptoms (AIMSS), including joint pain, stiffness, and myalgias, affect 20–74% of women with BC, with a pooled prevalence of 46% [125]. Addressing musculoskeletal symptoms is crucial not only for improving patient comfort but also for ensuring adherence to endocrine therapy. Indeed, discontinuation rates due to AIMSS can reach up to 30%, significantly compromising the efficacy of treatment and increasing the risk of BC recurrence [126].

Gupta et al. highlighted that there is no universally effective solution for managing AIMSS; however, integrating both pharmacological and non-pharmacological interventions through a multidisciplinary approach is crucial not only to alleviate symptoms but also to support sustained adherence and optimize long-term cancer outcomes [125,127].

Among non-pharmacological interventions, structured exercise has emerged as an effective strategy for managing AI-induced musculoskeletal symptoms in BC survivors. The phase III HOPE trial demonstrated that aerobic and supervised physical exercise alleviates AIMSS by 30% compared with the placebo group. In addition, participants allocated to the exercise group reported improvements in both joint pain and stiffness compared to usual care, as well as enhanced physical function [62]. Additional forms of physical activity, such as Nordic walking, aerobic programs, and aquatic exercises, have been assessed in smaller-scale studies with limited follow-up durations and have shown beneficial effects in reducing joint pain [63,128,129,130,131].

Yoga has emerged as a promising nonpharmacological intervention for managing AIMSS. In a pilot study by Jacobsen et al., participation in a structured Iyengar yoga program was associated with clinically meaningful reductions in joint pain severity and stiffness, as well as improvements in physical function and overall well-being [132]. These findings were supported by a subsequent trial conducted by Galantino et al., which demonstrated that a 6-week yoga intervention not only improved musculoskeletal symptoms but also enhanced functional outcomes, including balance, flexibility, and QoL [64].

Vitamin D supplementation has been investigated as a potential strategy for alleviating AIMSS given its anti-inflammatory properties. Although findings from randomized trials have been inconsistent, some data indicate that ensuring sufficient vitamin D levels or correcting deficiency may lead to modest improvements in joint pain and physical performance in this group of patients [66,67,68].

Concomitant therapies, such as omega-3 fatty acids and nonsteroidal anti-inflammatory drugs (NSAIDs), have been investigated for their potential to alleviate AIMSS-associated pain. In the SWOG S0927 trial, omega-3 supplementation showed promising results in mitigating joint pain and stiffness, but the differences with the placebo group were not statistically significant [70]. However, a subsequent analysis revealed that obese patients receiving omega-3 had significantly greater pain reduction than those receiving a placebo at 24 weeks [71]. Similarly, NSAIDs are frequently used in clinical practice to manage AI-associated arthralgias. Together, these agents may provide effective symptom control, particularly in patients who are not candidates for or have not responded adequately to lifestyle or exercise-based interventions.

In addition, duloxetine, an SNRI, has emerged as a pharmacological option for patients with persistent or severe AIMSS. A pilot trial conducted in 35 patients demonstrated a reduction of at least 30% in average pain levels after 8 weeks of therapy [79]. These findings are further reinforced by the results of the SWOG S1202 trial, which assessed the efficacy of duloxetine in managing joint symptoms caused by AI [65]. Specifically, the results demonstrated a clinically meaningful reduction in joint pain and stiffness within 6 weeks compared to placebo, associated with notable improvements in physical functioning and overall QoL, supporting its use as a therapeutic option in this patient population.

For many women who do not experience sufficient symptom relief from standard analgesics, acupuncture has demonstrated efficacy in reducing joint pain, as evidenced by the findings of a randomized, placebo-controlled trial [133,134]. In a large, randomized trial, Hershman et al. demonstrated a statistically significant improvement in pain scores with true acupuncture compared to sham acupuncture or usual care. This benefit persisted for 24 weeks, suggesting a sustained analgesic effect [135].

In cases of severe or refractory symptoms, modification of endocrine therapy may be necessary to improve tolerability and adherence. The phase III SOLE trial evaluated intermittent versus continuous letrozole administration as an extended adjuvant therapy in postmenopausal women with HR+ early BC. Although no significant difference in disease-free survival was observed between the two strategies, intermittent dosing was associated with less deterioration in patient-reported symptoms and overall QoL [136]. These findings suggest that intermittent letrozole may be a viable option for selected patients experiencing substantial treatment-related toxicity, offering improved tolerability without compromising its efficacy. Alternatively, switching to a different AI or tamoxifen has been associated with improved tolerability in some patients. In the ATOLL trial, 70% of patients continued endocrine therapy for over 6 months after switching to other AIs, suggesting that a subset of patients who switch between different AIs report a reduction in symptom burden without compromising the efficacy of endocrine treatment [126]. Similarly, the ELPh study showed that approximately two-thirds of patients who discontinue one AI due to side effects can continue treatment for at least six months after switching to another. This strategy offers a viable option for enhancing adherence in women experiencing intolerance to their initial AI. However, the reasons for the differing tolerability within the same patient remain unclear and merit further investigation [69].

### 3.3. Bone Health

Estrogen plays a crucial role in maintaining bone homeostasis by inhibiting osteoclast activity, stimulating osteoprotegerin expression, and promoting osteoblast survival. Its deficiency accelerates bone resorption, leading to an annual bone loss of 2–4%, approximately twice the rate observed in natural menopause [137]. These effects translate into a clinically meaningful increase in fracture risk; in the ATAC trial, anastrozole was associated with a 30% increase in fracture incidence compared with tamoxifen (11% vs. 7.7% at 5 years) [138]. Observational studies further underscore this risk, showing elevated hip and forearm fracture rates in AI users compared to those in tamoxifen-treated patients, particularly in those aged 50–65 years [139].

Both the ASCO and ESMO guidelines recommend baseline dual-energy X-ray absorptiometry for all postmenopausal women initiating AI therapy, followed by periodic reassessment [140]. Pharmacological intervention is advised for women with T-scores ≤–2.0 or those with T-scores between –1.5 and –2.0, plus additional risk factors such as age > 65 years, low BMI (<20kg/m^2^), family history of hip fracture, corticosteroid use, or smoking. Repeat dual-energy X-ray absorptiometry is generally recommended at 12 months or sooner if annual bone mineral density loss exceeds 5–10% [137,140].

Antiresorptive therapies, such as bisphosphonates and denosumab, are effective in mitigating AI-induced bone loss. Zoledronic acid (4 mg intravenously every 6 months) has shown consistent benefits in large randomized trials, stabilizing lumbar spine and hip bone mineral density, and reducing vertebral fractures over 3 years [72]. For instance, the Z-FAST and ZO-FAST studies demonstrated bone mineral density preservation and fracture risk reduction with immediate versus delayed zoledronic acid usage [30,73]. Denosumab (60 mg subcutaneous every 6 months) was evaluated in the ABCSG-18 trial involving over 3400 postmenopausal women on AIs, achieving a 50% reduction in clinical fracture risk sustained through 8 years of follow-up [74]. Moreover, the findings suggest that fracture risk among postmenopausal BC patients undergoing AI therapy occurs independently of baseline bone mineral density (BMD). Denosumab demonstrated similar efficacy in reducing fracture rates both in patients with normal BMD (T-score ≥ −1) and those with reduced BMD at treatment initiation. These results imply that factors beyond bone density may play a critical role in fracture risk. Other meta-analyses confirm that while both therapies improve bone mineral density, denosumab confers superior fracture risk reduction [75,141]. Moreover, the EBCTCG meta-analysis reported that bisphosphonate use in postmenopausal women reduced bone recurrence by 34% and BC-specific mortality by 17% [142].

However, the adverse effects of antiresorptive agents must be carefully considered. Bisphosphonates, while associated with rare occurrences such as osteonecrosis of the jaw and atypical femoral fractures, typically accumulate in bone and offer lingering protection post-treatment [72,142]. In contrast, denosumab requires continuous administration, and abrupt discontinuation can provoke rebound bone turnover and vertebral fractures [140]. Clinicians must weigh these risks when choosing the optimal agent and counsel the patients accordingly.

Other pharmacological and non-pharmacological strategies play complementary yet vital roles. A daily calcium intake of 1000–1200 mg and vitamin D supplementation (800–1000 IU/day) are standard to ensure adequate skeletal support [77]. Weight-bearing and resistance exercises, while proven to improve bone mineral density, lack conclusive evidence for fracture prevention in this population. Lifestyle measures, such as smoking cessation and minimizing alcohol intake, further enhance bone health [76,140].

Emerging strategies include monitoring bone turnover markers, such as C-terminal telopeptide and bone alkaline phosphatase, for the early detection of excessive bone resorption. While procollagen type I N propeptide and β-isomerized C-terminal telopeptide of type I collagen have been recognized as reference markers for treatment monitoring, they have not yet been incorporated into routine clinical decision-making due to analytical variability and insufficient validation in fracture risk algorithms [143,144].

### 3.4. Cognitive Changes and Mood Disorders

Cognitive changes and mood disturbances are frequently reported by BC survivors undergoing AI therapy, with prevalence rates reaching up to 51% for depression, 59% for insomnia, and 42% for anxiety in patients receiving exemestane with ovarian suppression [145]. Although often subtle, these symptoms can substantially impact QoL and adherence to long-term treatment.

These psychological disturbances may also exacerbate other treatment-related toxicities. A recent cross-sectional study demonstrated that anxiety was significantly associated with vaginal-related sexual health problems, suggesting that psychosocial interventions targeting anxiety may have broader utility in addressing sexual health concerns [146].

Despite their high prevalence and clinical impact, specific interventions and dedicated studies addressing the management of these symptoms in patients receiving AI remain largely lacking. Nonetheless, mind–body interventions, such as structured psychological support, relaxation techniques, and mindfulness, have demonstrated benefits in reducing anxiety and depression in BC survivors, offering a potentially valuable strategy even in this specific therapeutic context [77].

Electroacupuncture and sham acupuncture have been specifically evaluated in patients undergoing AI therapy. In a randomized controlled trial by Mao et al., electroacupuncture significantly improved fatigue, anxiety, and depression in BC survivors experiencing AI-related arthralgia compared with usual care [78]. By contrast, sham acupuncture had a significant effect only on depression. These findings suggest that electroacupuncture may serve as a beneficial non-pharmacological strategy for addressing psychological distress in this setting.

Pharmacological interventions should be considered in patients with persistent or severe symptoms that do not respond adequately to nonpharmacological strategies. Unlike tamoxifen, AIs are not significantly affected by drug–drug interactions with most antidepressants, which expands the range of therapeutic options available. As outlined in the recent ASCO guidelines, the decision to initiate pharmacological treatment must be individualized, considering symptom burden, patient preference, and overall risk–benefit profile [147].

Among pharmacological treatment options, duloxetine has been investigated in a pilot study and demonstrated improvements in mood disturbances, including depression and sleep-related symptoms, in patients undergoing AI therapy [79]. In particular, a starting dose of 30 mg/day for 7 days, followed by 60 mg/day for 21 days, resulted in a significant reduction in depression. Generally, despite specific data on other SSRIs and SNRIs in this population being limited, evidence from general BC cohorts supports their tolerability. For instance, escitalopram co-prescription did not alter estradiol levels in postmenopausal patients on anastrozole, and the drug showed no significant pharmacokinetic interaction [148].

### 3.5. Gynecological and Sexual Dysfunction

Although essential for tumor suppression, the estrogen deprivation inherent in AI therapy frequently causes gynecologic and sexual adverse effects, including vaginal dryness, dyspareunia, diminished libido, reduced sexual satisfaction, and psychological distress, all of which can profoundly impact patients’ QoL and treatment adherence [149]. In the SOFT and TEXT trials, which evaluated adjuvant endocrine strategies in premenopausal women with HR+ BC, 53.7% of patients receiving exemestane with ovarian suppression reported vaginal dryness, 45.6% experienced decreased libido, and 31.6% reported dyspareunia [145]. These findings underscore the profound impact of estrogen deprivation on sexual function and overall QoL in this population group. A recent study also highlighted that a significant proportion of oncologists do not routinely inquire about these side effects and that, in most cases, patients themselves do not seek help for managing such symptoms [150]. Changes in the vaginal microbiome and vaginal wall thickness have also been documented in prospective studies. In one study, transvaginal ultrasonography revealed measurable differences in vaginal wall thickness in patients receiving AIs [151]. Furthermore, a separate prospective trial comparing the vaginal microbiome of patients with BC undergoing AI therapy with that of healthy volunteers identified significant alterations in the microbial composition [152].

In response to symptoms related to estrogen deprivation, several pharmacological and non-pharmacological interventions have shown promise in alleviating symptoms and improving adherence to AI therapy, although none of the currently available strategies for managing these symptoms are officially approved for clinical practice due to limited evidence.

Non-pharmacological therapies should be considered the first-line approach for managing AI-related gynecologic symptoms, especially given the current lack of robust long-term data on the safety of pharmacological options in patients with BC [9]. A randomized pilot study demonstrated the efficacy of a multi-component combined intervention, including vaginal gels, dilator therapy, and psychosexual counseling, in preventing gynecologic and sexual dysfunction in patients undergoing adjuvant AI therapy [153]. The results showed that participants who received the intervention had significantly better sexual function scores and lower levels of sexual distress than the control group at 6 months. Importantly, adherence to AI therapy remained high, suggesting that proactive management of genitourinary symptoms may support treatment continuity and underscores the value of early multidisciplinary supportive care strategies to mitigate sexual side effects and preserve QoL in BC survivors.

A systematic review by Cyr et al. assessed the impact of pelvic floor muscle training and education-based interventions on pelvic, sexual, and psychological health outcomes in women treated for BC [154]. These findings suggest that non-pharmacologic approaches can improve urinary incontinence, vaginal symptoms, sexual dysfunction, and pelvic floor muscle performance. Some studies have also reported enhancements in psychological outcomes, including reduced anxiety and improved body image, highlighting the potential role of pelvic rehabilitation and educational support in comprehensive survivorship care for AI-treated patients.

Among non-pharmacological strategies, non-ablative CO_2_ laser therapy has demonstrated improvements in vulvovaginal atrophy symptoms and sexual function in BC survivors. In a prospective trial by Lami et al., postmenopausal women with a history of BC underwent three fractional CO_2_ laser sessions [80]. The study reported significant improvement in both subjective and objective measures of genitourinary syndrome of menopause, with a 42% reduction in the Vaginal Health Index in women with baseline severe vulvovaginal atrophy and less marked improvement in those with mild symptoms. Patients experienced reductions in vaginal dryness, dyspareunia, and burning sensations, accompanied by enhanced sexual function and QoL. These findings were complemented by a double-blind, randomized, sham-controlled trial by Cruff et al., which further evaluated the efficacy of fractional CO_2_ laser therapy [81]. While both the laser and sham groups experienced improvements in symptoms over time, the study did not demonstrate a statistically significant difference between the groups, suggesting a potential placebo effect or the need for refined patient selection criteria. Overall, while non-ablative CO_2_ laser therapy appears to be a safe and well-tolerated option, further large-scale randomized trials are required to confirm its efficacy and establish its role in clinical practice settings. Another type of laser, the non-ablative solid-state vaginal laser (SSVL), has been evaluated in a multicenter, single-arm, non-randomized pilot study involving patients with BC after completion of adjuvant therapy with AIs [155]. The intervention demonstrated promising results, with clinical improvement in genitourinary symptoms, including vaginal dryness, irritation, and dyspareunia. According to Lubián-López et al., patients reported statistically significant improvements in both the Vaginal Health Index and Female Sexual Function Index after three SSVL treatment sessions. The therapy was well tolerated, with no significant adverse events, and the participants reported a high degree of satisfaction. These preliminary findings suggest that SSVL may offer a safe and effective non-hormonal alternative for managing vulvovaginal atrophy in HR+ BC survivors.

Vaginal oxygenation combined with hyaluronic acid is an emerging non-pharmacological technique currently under evaluation, which has shown improvements in vaginal atrophy, sexual function, and urinary symptoms [82]. Specifically, hyaluronic acid-based moisturizers appear to be safe, non-estrogenic options for managing genitourinary symptoms in patients with BC, particularly those undergoing AI therapy. A prospective, single-arm trial conducted by Carter et al. evaluated the safety and efficacy of a non-hormonal vaginal moisturizer containing hyaluronic acid [83]. Over a 12-week treatment period, the study demonstrated a significant and progressive reduction in vaginal dryness. Approximately 97% of the participants reported mild-to-severe dryness at baseline, which decreased to 45% after three treatment cycles and further to 26% at the final follow-up. Improvements were also observed in vulvar dryness, irritation, and sexual function, with the product being well tolerated and no serious adverse events reported.

Despite the effectiveness of non-pharmacological interventions, some patients may require additional therapeutic support for adequate symptom relief. In this context, local hormone therapy, particularly low-dose vaginal estrogen formulations, is considered the most effective option for managing the symptoms of genitourinary syndrome [156]. However, its use in patients with BC, especially those receiving AIs, remains controversial because of concerns regarding systemic absorption and potential interference with endocrine therapy. A preliminary study by Biglia et al. assessed the impact of low-dose vaginal estrogen therapy and non-hormonal moisturizers on serum estradiol levels in BC survivors with urogenital atrophy [86]. Their findings revealed that low-dose vaginal estrogen treatment was generally well tolerated, with no significant difference in circulating estrogen levels. These results support the hypothesis that carefully selected local estrogen therapies may not substantially compromise estrogen suppression in AI-treated patients. Further supporting this, a large retrospective cohort study by McVicker et al. examined the survival impact of vaginal estrogen therapy in 49237 BC survivors [87]. The study found no association between the use of vaginal estrogen and an increased risk of all-cause or BC-specific mortality in patients undergoing AI therapy. However, data on oncological safety remain limited. A nested case–control study by Le Ray et al. investigated the association between local estrogen therapy and BC recurrence in HR+ patients [88]. Although no significant increase in recurrence risk was observed, the study emphasized caution, particularly in patients concurrently receiving systemic endocrine therapy. Given the heterogeneity in formulations, dosages, and patient responses, large-scale prospective studies are necessary to clarify the long-term safety of vaginal estrogen in this population. Until more evidence is available, clinical guidelines suggest an individualized risk-benefit assessment and close monitoring when considering local estrogen interventions in HR+ BC survivors [89,116].

Ospemifene, a selective estrogen receptor modulator, has emerged as a potential non-estrogenic treatment option for vulvovaginal atrophy, particularly in postmenopausal women who are not suitable candidates for local estrogen therapy [90]. A retrospective study by Cai et al. examined data from clinical trials and post-marketing surveillance in patients with a history of BC [91]. The analysis reported no increased incidence or risk of BC recurrence among ospemifene-treated individuals with vulvovaginal atrophy, suggesting a favorable risk-benefit balance. Although ospemifene is not currently approved for use in women with active or prior HR+ BC because of the lack of prospective trials in this specific population, these findings provide reassuring safety signals that may justify further investigation.

Topical testosterone is a promising treatment option for managing sexual dysfunction. Evidence from a prospective study by Taranto et al. demonstrated that daily application of low-dose topical testosterone significantly improved vaginal atrophy and sexual symptoms [84]. Notably, the treatment did not result in any significant increase in circulating estradiol levels, suggesting that it may be a safe alternative for patients in whom estrogen-based therapies are contraindicated.

Finally, topical lidocaine has shown promise as a practical and effective intervention for dyspareunia. In a randomized controlled trial conducted by Goetsch et al., the application of aqueous 4% lidocaine to the vulvar vestibule significantly reduced pain during intercourse compared to standard care, which was associated with reduced sexual distress [85]. The study emphasized that lidocaine offers a safe, non-hormonal option that is particularly well-suited for estrogen-deprived patients, providing immediate symptom relief without systemic absorption or interference with ongoing endocrine therapy.

### 3.6. Fatigue

Fatigue is a frequently reported and distressing side effect among BC survivors undergoing AI therapy, with prevalence rates ranging from 30% to 90%, depending on the assessment methods and population characteristics [92]. Recent studies have reported even higher prevalence, with up to 86% of patients treated with AI experiencing fatigue and over half reporting moderate to severe symptoms [157].

Among non-pharmacological strategies, physical exercise has emerged as one of the most effective interventions for alleviating cancer-related fatigue in BC survivors [93]. Recent evidence indicates that exercise is particularly effective when initiated during and after active cancer treatment, supporting its role across the continuum of survivorship care. Both aerobic and resistance training have demonstrated significant benefits in reducing fatigue severity, enhancing physical function, and improving overall QoL during and after AI therapy [92,93]. A meta-analysis of randomized controlled trials confirmed that exercise interventions not only alleviate fatigue but also positively influence anxiety, depression, and aerobic capacity in women receiving endocrine treatment [94].

Mind–body therapies, such as yoga and meditation, have shown promise in clinical trials [95]. Yoga has emerged as a promising adjunctive intervention for managing cancer-related fatigue. In addition to improving physical function, flexibility, and sleep quality, yoga incorporates mindfulness and breathing techniques that may address psychological components of fatigue. A previous meta-analysis demonstrated that yoga significantly reduces fatigue in patients with BC, particularly when it is practiced consistently over several weeks [96].

CBT has emerged as an evidence-based, non-pharmacological intervention for managing cancer-related fatigue in BC survivors, including those receiving AI therapy [97,98]. Several randomized controlled trials have demonstrated that CBT significantly reduces fatigue intensity and fatigue-related functional impairment in cancer survivors, with sustained effects lasting for months after the intervention [99]. Notably, CBT appears particularly beneficial for patients with chronic fatigue and coexisting symptoms such as insomnia, depression, or anxiety, which commonly overlap with endocrine therapy-related side effects [158].

Recent evidence supports self-administered acupressure as a cost-effective and accessible non-pharmacological option for managing persistent cancer-related fatigue in BC survivors. A randomized clinical trial by Zick et al. suggested that these benefits were largely sustained at week 10, indicating a durable response following the cessation of the intervention [100]. Notably, compared to stimulating acupressure, relaxing acupressure also produced significant improvements in sleep quality and multiple QoL domains, such as somatic symptoms, fitness, and social support.

In addition to non-pharmacological strategies, pharmacological and nutraceutical interventions have been investigated for their potential to alleviate fatigue in BC survivors. Despite psychostimulants such as methylphenidate and modafinil showing modest efficacy in select populations, their use remains limited because of inconsistent results across trials and concerns regarding side effects and long-term safety [102]. Moreover, hematopoietic agents, such as erythropoiesis-stimulating agents, have demonstrated some benefits in patients with anemia-related fatigue but are generally not recommended outside this context due to potential risks, including thromboembolic events [103]. A recent meta-analysis by Yennurajalingam et al. explored the efficacy of a broad range of pharmacological, nutraceutical, and phytopharmaceutical interventions [101]. The analysis highlighted that ginseng, L-carnitine, and coenzyme Q10 may offer mild to moderate improvements in fatigue, although the overall quality of evidence was variable and often limited by small sample sizes and methodological heterogeneity of the studies. Notably, they emphasized that no single agent has consistently demonstrated robust clinical benefits across diverse cancer populations, underscoring the need for individualized approaches and further high-quality trials to clarify the role of these interventions in managing fatigue among BC survivors.

**Figure 2:**
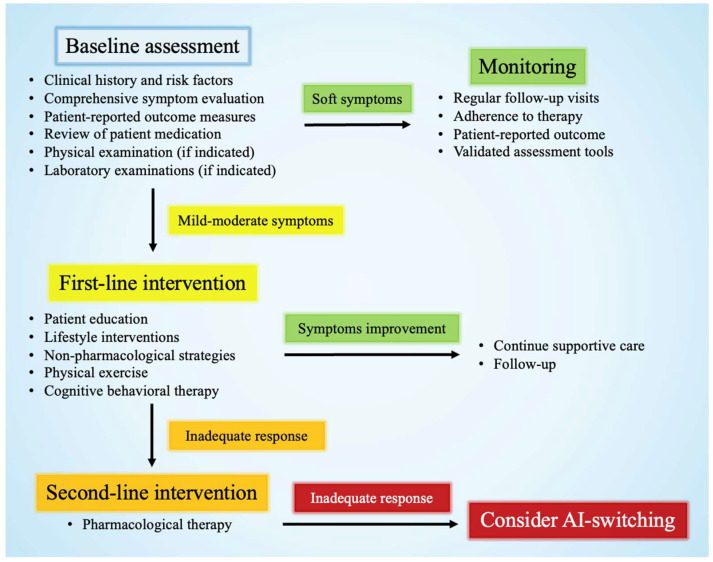
Stepwise management approach based on symptom severity.

In conclusion, the effective management of AI-related toxicities is critical to maintaining treatment adherence and optimizing long-term outcomes in patients with HR+ BC. Considering the various and often overlapping nature of these toxicities, clinicians are encouraged to implement a comprehensive, individualized care model that combines medical and supportive approaches (Figure 2). Interventions should be tailored to each individual’s specific symptom burden, comorbidities, and personal preferences, in order to improve QoL but also reinforce the overall success of endocrine therapy in the survivorship setting.

## 4. Addressing Cardiovascular Risks

### 4.1. Cardiovascular Complications

BC survivors face an elevated risk of CV disease, attributable not only to shared risk factors but also to the effects of endocrine therapy. Estrogen depletion induced by AIs may negatively impact the CV system, prompting concerns regarding myocardial infarction (MI) and heart failure (HF), and arrhythmias in this population [39].

(a) Hypertension. Estrogens are known to modulate the renin-angiotensin and nitric oxide (NO) pathways, and their depletion can cause vasoconstriction, hypertension, and left ventricular remodeling. Several clinical studies have reported increases in both systolic and diastolic blood pressure during AI therapy [159]. A large observational cohort found a 15–20% higher risk of new-onset hypertension during AI treatment compared with baseline, underscoring the need for vigilant monitoring [160].

(b) Ischemic heart disease. AI therapy has been associated with an increase in ischemic cardiac events, including MI. In a meta-analysis of 25 studies, approximately 6% of AI-treated patients experienced a CV event during follow-up, with ischemic heart disease being among the most common (approximately 3.8 per 100 patients) [161]. Population-based data support a higher incidence of MI in AI users; one large cohort study found that the MI rate in AI-treated women was roughly double that of those on other endocrine therapies (3.9 vs. 1.8 per 1000 person-years). Though some analyses did not reach statistical significance for MI risk, the trend toward increased incidence with AIs has been consistently observed. Notably, an extended duration of AI use may compound this risk; women treated with AIs for ≥4 years had over twice the risk of acute ischemic heart disease compared to those with shorter exposure [162]. These findings underscore the fact that AIs can contribute to coronary artery disease and MI in survivors.

(c) Heart failure. HF is another concern, often resulting from previous ischemic damage or cardiomyopathy. In the meta-analysis by Yoo et al., approximately 2.1% of patients treated with AI developed HF during the study period [161]. Similarly, in a UK population-based cohort, AI therapy was significantly associated with higher HF incidence (5.4 vs. 1.8 per 1000 person-years) and CV mortality risk (HR 1.50) compared to tamoxifen. While direct comparisons to tamoxifen highlight the difference, these data indicate that AIs can contribute to HF development, particularly in patients with underlying coronary disease or those receiving prolonged therapy [163]. Mechanistically, the elevation in MI risk with AIs may partly explain the parallel increase in HF, as ischemic cardiomyopathy is a leading cause of HF. Some have hypothesized that metabolic changes induced by estrogen depletion could promote HF over time [164]. Although the absolute risk of HF with AIs is modest, it should not be overlooked, especially in elderly patients or those receiving extended AI therapy.

(d) Atrial fibrillation. Emerging evidence suggests that AIs contribute to arrhythmogenic risk, particularly atrial fibrillation (AF). The cardioprotective effects of estrogen include stabilization of cardiac electrophysiology and autonomic tone, partially through enhancement of NO release and ion channel modulation [165,166]. In a large propensity-matched cohort, AF occurred in 0.5% of AI users within one year (versus 0.4% on tamoxifen), and the risk remained elevated at 5 years (1.2% vs. 1.1%, RR ~1.13) [167], consistently also in ethnic groups traditionally poorly represented in clinical studies [168]. Long-term AI use may further increase arrhythmia risk: treatment for more than 4 years of AI therapy was associated with a 2.12-fold higher hazard of arrhythmia [162].

(e) Dyslipidemia and metabolic effects of the drug. Unlike tamoxifen, which improves lipid profiles via partial estrogen receptor agonism, AIs often worsen the lipid profiles. Beyond lipids, AI-induced estrogen deprivation may promote other features of metabolic syndrome [169], including insulin resistance and diabetes, thereby compounding CV risk [170]. In a cohort of nearly 9000 BC survivors, AIs were associated with substantial development of cardiometabolic risk factors over ~8 years, even if partially impacted by aging and baseline risks [170]. Similar data were obtained from a previous meta-analysis [171]. Comparative studies indicate that exemestane increases LDL/HDL ratios and apolipoprotein B/A1, whereas anastrozole and letrozole increase triglyceride levels more significantly [172].

Notably, elevated CV risks have also been documented in premenopausal women undergoing ovarian suppression combined with AI therapy. One study found that women under 55 years had a CV event rate of 2.3 per 100 person-years on AI plus suppression, compared to 1.0 on tamoxifen [173].

### 4.2. Monitoring and Management Strategies

During AI therapy, CV evaluation should include defined action points starting from the baseline visit and continuing during the follow-up to minimize CV risk and enable early detection of complications (Table 2).

(a) Baseline CV Risk Assessment. Before initiating AI therapy, each patient’s CV risk profile should be assessed carefully. This involves reviewing the history of CV disease and traditional risk factors, such as hypertension, diabetes, dyslipidemia, smoking, obesity, and family history. A baseline lipid panel, blood pressure measurement, and electrocardiography are indicated. In the case of a high or very high cardiovascular risk profile, echocardiography may be warranted [174].

(b) Lifestyle modification. Patients starting AIs should be counseled on heart-healthy lifestyle practices. Regular exercise, a balanced diet, weight management, and smoking cessation can significantly reduce the risk of CV disease [175]. Given the tendency of AIs to worsen metabolic parameters, dietary adjustments to counteract increases in cholesterol and blood sugar levels are prudent. Exercise can help improve HDL and vascular function, potentially offsetting some of the effects of AI [176].

(c) Periodic monitoring and surveillance are essential. Ongoing AI therapy requires regular monitoring of key cardiovascular parameters, particularly blood pressure, which should be assessed every 3–6 months, especially during the first year. Lifestyle modification should be emphasized. Annual ECG in women is reasonable, given the elevated AF risk, to enable timely intervention [177].

(d) Risk factor management. If patients develop or have pre-existing cardiovascular risk factors, clinical management is mandatory. For dyslipidemia, lifestyle measures may suffice for mild cases, but many patients will benefit from pharmacotherapy to control LDL cholesterol levels [178]. Antihypertensive medications should be initiated according to general cardiology guidelines if persistent hypertension develops [179].

(e) Individualized therapy. In the case of CV complications, the preferred approach is to treat the cardiac condition aggressively and continue AI therapy, given that AIs improve BC survival [5]. Decisions about extending AI therapy beyond the standard 5 years should also be discussed in light of the patient’s CV risk profile and potential complications that may develop during the follow-up period, even if contrasting results are reported about cardiovascular risk for long-term treatment [180]. In some cases, switching endocrine therapy to tamoxifen may be considered if cardiovascular side effects are thought to be directly related to AIs and are severe, outweighing the oncological benefits.

In summary, there is no evidence that AIs directly cause cardiomyocyte apoptosis or acute cardiomyopathy in a toxic manner. Instead, their detrimental effects can be considered indirect and chronic, involving the acceleration of traditional risk factors and the removal of estrogen-mediated cardiac protection. Collaboration between oncologists and cardiologists is necessary, and some scientific societies have suggested the term cardio-oncology specialists, indicating a physician with competence in both fields [181]. The focus must always ensure that cancer survivors not only live longer due to effective therapies but also enjoy a heart-healthy life during and after the treatment.

## 5. Expert Opinion and Future Perspectives

In light of the evidence summarized in Table 3 and the updated literature reviewed in this work, it is evident that the toxicity profile of AIs has been extensively characterized in previous studies, yet significant gaps remain in translating these findings into consistent, patient-centered clinical practice. While previous reviews have provided comprehensive overviews of individual symptom domains, our analysis integrates updated trial data, prioritization of interventions, and explicit grading of evidence to support practical decision-making. This combined approach aims to bridge the gap between clinical evidence and real-world applicability, with a focus on long-term survivorship and individualized care.

From a critical standpoint, current management of AI-induced toxicities still suffers from a fragmented approach: robust, evidence-based recommendations exist for some domains, such as bone health and vasomotor symptoms, but remain scarce or inconsistent for others, including cognitive impairment and sexual dysfunction. Many interventions with promising preliminary results, such as acupuncture for AIMSS or vaginal laser therapy for genitourinary syndrome, remain investigational due to limited high-quality data. Furthermore, the translation of clinical trial findings into daily practice is hindered by heterogeneity in patient populations, limited follow-up, and variability in how toxicities are assessed and reported.

From our expert perspective, the next leap forward in adjuvant endocrine therapy lies in a more personalized, anticipatory, and integrated model of care. This means identifying patients at higher risk for specific toxicities early on (potentially using biomarkers, patient-reported outcomes, or digital monitoring) and intervening proactively rather than reactively. For example, Conte et al. evaluated how single-nucleotide polymorphisms may be linked to skeletal and cardiovascular events, highlighting how pharmacogenomics could help tailor endocrine strategies [182]. It also means acknowledging that what matters most to patients is not only preventing recurrence, but also preserving daily QoL, physical function, cognitive integrity, and sexual health throughout years of therapy.

There is also a growing interest in how survivorship care can be made more structured and multidisciplinary. In particular, collaborations between oncologists, physiatrists, psychologists, gynecologists, cardiologists, and general practitioners could help address the full spectrum of AI-induced adverse effects. Integrating physical activity programs, non-hormonal interventions, psychological support, and even lifestyle coaching into oncology pathways is no longer optional; rather, it is increasingly recognized as good clinical practice.

Looking ahead, one of the most promising frontiers is the development of novel agents that could retain or even enhance anti-tumor efficacy while improving tolerability. Among these, oral selective estrogen receptor degraders (SERDs) are generating significant interest. While initially developed for advanced disease, they are now being evaluated in early-stage settings: elacestrant (NCT06492616), camizestrant (NCT05774951, NCT05952557), imlunestrant (NCT05514054), and giredestrant (NCT04961996). These agents act by directly degrading the estrogen receptor, potentially overcoming mechanisms of endocrine resistance. Importantly, their distinct toxicity profiles could be considered alongside patient preferences and comorbidities, making them a valuable option for those who are especially vulnerable to AI-related side effects. However, their precise role in the adjuvant setting remains to be defined.

These trials also reflect a broader conceptual shift in breast cancer survivorship: away from a one-size-fits-all approach and toward personalized strategies that reduce recurrence risk while maintaining QoL. It is conceivable that, shortly, molecular markers such as ESR1 mutations, pharmacogenetic profiles, or patient-reported symptom patterns will guide the choice of adjuvant endocrine therapy. In this context, oral SERDs could represent not only an alternative but a tailored solution for patients with a specific risk–benefit profile.

Ultimately, the success of adjuvant endocrine therapy will be measured not only in disease-free survival but also in the ability to sustain treatment without compromising the lived experience of patients. In this sense, toxicity is not a collateral event; it is a central determinant of therapeutic success.

## 6. Conclusions

AIs remain a cornerstone of adjuvant endocrine therapy for HR+ early breast cancer, offering proven benefits in reducing recurrence and improving survival. However, prolonged use, often beyond five years, can lead to a broad spectrum of toxicities, including musculoskeletal symptoms, vasomotor disturbances, bone loss, cognitive changes, genitourinary symptoms, cardiovascular complications, and fatigue. These side effects can compromise QoL and adherence, making early recognition and proactive management essential to preserve oncologic benefit.

While some areas, such as bone health and vasomotor symptoms, are supported by robust evidence, management strategies for cognitive and sexual dysfunction remain limited. Several promising interventions, including acupuncture for AIMSS or vaginal laser therapy, are still investigational due to insufficient high-quality data. The challenge is to integrate trial findings into daily practice, despite heterogeneous populations, limited follow-up, and variability in toxicity assessment.

A more personalized and anticipatory model of survivorship care is needed, combining early risk identification, through biomarkers, pharmacogenetics, and patient-reported outcomes, with proactive, multidisciplinary strategies. Novel agents, such as oral selective estrogen receptor degraders, may soon expand options for patients vulnerable to AI-related side effects.

Ultimately, the goal is not only to extend survival but to ensure it is matched by sustained QoL, autonomy, and comfort. In this context, toxicity is not a collateral issue: it is a central determinant of therapeutic success.

## Figures and Tables

**Table 1:** Principal side effects and management during aromatase inhibitor therapy, according to recommendation level and evidence strength.

Side Effect	Intervention	Recommendation Level	Evidence Strength	Key References
Vasomotor symptoms	CBT	First-line	High	Mann et al., 2012 [42]; Duijts et al., 2012 [43]
	Acupuncture	First-line	High	Wang et al., 2018 [44]; Zhang et al., 2025 [45]; Chien et al., 2017 [46]; Yuanqing et al., 2020 [47]; Walker et al., 2010 [48]
	Venlafaxine, gabapentin, clonidine	First-line	High	Loprinzi et al., 2000 [49]; Carpenter et al., 2007 [50]; Buijs et al., 2009 [51]; Pandya et al., 2005 [52]
	Lifestyle modification, dietary interventions, weight loss	Second-line	Moderate	Marsden et al., 2019 [53]; Su et al., 2010 [54]; Thurston et al., 2009 [55]
	Fezolinetant, elinzanetant	Second-line	Moderate	Lederman et al., 2023 [56]; Cardoso et al., 2025 [57]
	Hypnosis	Second-line	Moderate–low	Elkins et al., 2008 [58]; MacLaughlan David et al., 2013 [59]
	SGB	Second-line	Moderate–low	Haest et al., 2012 [60]; Rahimzadeh et al., 2018 [61]
Musculoskeletal symptoms	Exercise, yoga	First-line	High	Irwin et al., 2015 [62]; Bender et al., 2025 [63]; Galantino et al., 2012 [64]
	Duloxetine	First-line	High	Henry et al., 2018 [65]
	Vitamin D	Second-line	Moderate	Rastelli et al., 2011 [66]; Khan et al., 2017 [67]; Shapiro et al., 2016 [68]
	Switch AI	Second-line	Moderate	Kadakia et al., 2016 [69]
	Omega−3	Second-line	Low	Hershman et al., 2015 [70]; Shen et al., 2018 [71]
Bone health	Bisphosphonates (Zoledronic acid)	First-line	High	Coleman et al., 2013 [30]; Hines et al., 2009 [72]; Brufsky et al. 2006 [73]
	Denosumab	First-line	High	Gnant et al., 2015 [74]; Abdel-Rahman et al., 2016 [75]
	Calcium, vitamin D	First-line	High	Coleman et al., 2020 [76]
Cognitive changes and mood disorders	Psychological therapy	Second-line	Moderate	Jassim et al., 2023 [77]
	Electroacupuncture	Second-line	Moderate	Mao et al., 2014 [78]
	Duloxetine	Second-line	Low	Herny et al., 2011 [79]
Gynecological and sexual disfunction	CO2 laser	Second-line	Moderate	Lami et al., 2024 [80]; Cruff et al., 2021 [81]
	Vaginal oxygenation and hyaluronic acid	Second-line	Moderate	Massarotti et al., 2023 [82]; Carter et al., 2021 [83]
	Topical testosterone	Second-line	Moderate	Taranto et al., 2024 [84]
	Topical lidocaine	Second-line	Moderate	Goetsch et al., 2015 [85]
	Local estrogen	Investigational	Moderate	Biglia et al., 2010 [86]; McVicker et al., 2024 [87]; Le Ray et al., 2024 [88]; Faltinová et al., 2025 [89]
	Ospemifene	Investigational	Moderate	Portman et al., 2013 [90]; Cai et al., 2020 [91]
Fatigue	Physical exercise	First-line	High	Hagstrom et al., 2016 [92]; Baumann et al., 2017 [93]; Cramp et al., 2012 [94]
	Yoga, meditation, CBT	First-line	High	Cramer et al., 2015 [95]; Hou et al., 2024 [96]; Hosseini Koukamari et al., 2025 [97]; Poort et al., 2020 [98]; Gielissen et al., 2007 [99]
	Acupressure	First-line	High	Zick et al., 2016 [100]
	Ginseng, L-carnitine, Coenzyme Q10	Second-line	Low	Yennurajalingam et al., 2022 [101]
	Psychostimulants	Investigational	Moderate	Andreas et al., 2023 [102]
	Hematopoietic agents	Investigational	Moderate	Bohlius et al., 2014 [103]

**Legend**: Recommendation level: *first-line:* supported by multiple studies or recommended by clinical guidelines. *Second-line:* effective in selected cases but with weaker or variable evidence. *Investigational:* based on preliminary data, not recommended for routine use. Evidence strength: qualitative rating of the supporting evidence: *High* = consistent, robust clinical data (e.g., multiple randomized trials or high-quality meta-analyses); *Moderate* = some clinical evidence but with limitations (small trials, indirect data); *Low* = limited, conflicting, or mainly expert opinion. Abbreviations: CBT, cognitive behavioral therapy; SGB, stellate ganglion block.

**Table 2:** Cardiovascular monitoring strategies.

	Baseline	Frequency	Intervention
CV risk assessment	All patients		
Complete lipid profile	All patients	Annually	Dietary +/− pharmacological
Blood pressure measurement	All patients	Every 3–6 months	Lowering pharmacological according to general population threshold
Lifestyle modification counseling	All patients	Every 3–6 months	
Electrocardiogram	All patients	Annually	Timely atrial fibrillation treatment, including anticoagulation and rhythm and/or rate control
Echocardiogram	High or very high CV risk	According to CV diagnosis	Referral to cardiologist
Carotid ultrasound	High or very high CV risk	According to CV diagnosis	Referral to vascular surgeon

**Legend:** CV, cardiovascular.

**Table 3:** Principal studies evaluating the management of aromatase inhibitor-related toxicities.

Study	AI Therapy	Study Type	Key Endpoint	Main Toxicity	Key Findings
Walker et al., 2010 [48]	Anastrozole	RCT	Hot flashes frequency	VMS	Acupuncture matched venlafaxine in reducing hot flash frequency
Loprinzi et al., 2000 [49]	Mixed	RCT	Hot flashes frequency and severity	VMS	Venlafaxine alleviates hot flashes; 75 mg daily is optimal.
Su et al., 2010 [54]	Mixed	Cross-sectional survey	Presence of hot flashes	VMS	Weight gain is linked to hot flash risk
Duijts et al., 2012 [43]	Mixed	RCT	Endocrine symptoms	VMS	CBT and/or PE improved menopausal symptoms
Cardoso et al., 2025 [57]	Mixed	RCT	Hot flashes frequency	VMS	Elinzanetant significantly reduced VMS frequency
Rastelli et al., 2011 [66]	Anastrozole	RCT	Pain reduction	AIMSS	Vitamin D has a beneficial effect on musculoskeletal pain
Briot et al., 2010 [126]	Anastrozole → letrozole	Prospective non-randomized	Percentage of discontinuation of letrozole due to AIMSS	AIMSS	Switching between AIs enabled 70% of patients to continue treatment >6 months
Crew et al., 2010 [134]	Mixed	RCT	Pain reduction	AIMSS	True acupuncture reduced joint pain and stiffness vs. sham acupuncture
DeNysschen et al., 2014 [129]	Mixed	Pilot	AIMSS2 scale variation	AIMSS	Home-based exercise reduced joint pain
Irwin et al., 2015 [62]	Mixed	RCT	Pain score	AIMSS	Exercise reduced AI-related arthralgia pain scores by approximately 30%
Kadakia et al., 2016 [69]	Mixed	Prospective	Adherence	AIMSS	Switching AI allowed 2/3 of patients to continue therapy
Henry et al., 2018 [65]	Mixed	RCT	Pain reduction	AIMSS	Duloxetine significantly improved pain vs. placebo
Hershman et al., 2018 [135]	Mixed	RCT	Pain reduction	AIMSS	True acupuncture significantly reduced joint pain vs. sham
Bender et al., 2025 [63]	Mixed	RCT	Pain reduction	AIMSS	Aerobic exercise prevents pain increase
Mao et al., 2014 [78]	Mixed	RCT	Pain score, fatigue, psychological distress	AIMSS, fatigue, mood changes	Electro-acupuncture improved fatigue, anxiety, and depression in BC patients who experienced arthralgia related to AI use
Gnant et al., 2015 [74]	Anastrozole, Letrozole	RCT	Fracture risk	Bone loss	Denosumab reduced the rate of clinical fractures.
Coleman et al., 2013 [30]	Letrozole	RCT	BMD change	Bone loss	Zoledronate preserved BMD and is associated with improved DFS vs. letrozole alone.
Advani et al., 2017 [153]	Mixed	Pilot study	FSFI score	Gynecological symptoms	Active intervention resulted in better outcomes at 6 months
Carter et al., 2021 [83]	Mixed	Prospective	VAS and VuAS changes	Gynecological symptoms	HLA moisturization improved vulvovaginal health/sexual function
Lubián-López et al., 2023 [155]	Mixed	Pilot study	Vulvo-vaginal atrophy	Gynecological symptoms	Non-ablative SSVL improved vaginal atrophy, vaginal pH, dyspareunia, and sexual function
Taranto et al., 2024 [84]	Mixed	Pilot study	Serum estradiol elevation, sexual function improvement	Gynecological symptoms	Topical testosterone seems to be safe and effective in improving sexual function
Faltinová et al., 2025 [89]	Letrozole	Prospective	Changes in serum E2 levels, menopausal symptoms	Gynecological symptoms	Intravaginal estradiol therapy during adjuvant letrozole resulted in transient increases in systemic E2 levels
Hagstrom et al., 2016 [92]	Mixed	RCT	Fatigue	Fatigue	16 weeks of high intensity RT significantly improved upper and lower body strength, and reduced perceived fatigue
Zick et al., 2016 [100]	Mixed	RCT	Change in fatigue score	Fatigue	Acupressure reduced fatigue and improved sleep quality and quality of life

Legend: AI, aromatase inhibitors; mixed, include multiple AIs or unspecified AIs; RCT, randomized control trial; VMS, vasomotor symptoms; AIMSS, aromatase inhibitor-induced musculoskeletal symptoms; BMD, bone mineral density; FSFI, female sexual function index; SSVL, solid-state vaginal laser; VAS, vaginal assessment scale; VuAS, vulvar assessment scale; HLA, hyaluronic acid; E2, estradiol; RT, resistance training.
